# Positive Self-Disclosure on Social Network Sites and Adolescents’ Friendship Quality: The Mediating Role of Positive Feedback and the Moderating Role of Social Anxiety

**DOI:** 10.3390/ijerph20043444

**Published:** 2023-02-15

**Authors:** Lizhong Liu, Tianyi Zhang, Lei Han

**Affiliations:** 1 School of Education, Central China Normal University, Wuhan 430079, China; 2 School of Psychology, Shandong Normal University, Jinan 250014, China

**Keywords:** positive self-disclosure, positive feedback, friendship quality, social anxiety, adolescence

## Abstract

In the current information age, SNSs (Social Network Sites) have been popular among young adolescents, and have also become a main manner to maintain social relationships. Against this background, based on relevant evidence, the present study aimed to examine the association between positive self-disclosure on SNSs and adolescents’ friendship quality, as well as the underlying mechanism—the potential mediating role of perceived positive feedback and the moderating role of social anxiety. A sample of 1713 adolescents aged 11 to 19 was recruited to participate in this study, to complete a set of scales. Results indicated that positive self-disclosure on SNSs was positively associated with adolescents’ friendship quality, and positive feedback significantly mediated the association between self-disclosure positivity and friendship quality. This mediating effect, moderated by social anxiety, could significantly moderate the mediating effect of positive feedback; specifically, compared with higher social anxiety adolescents, the association between positive self-disclosure and positive feedback was stronger among individuals with lower social anxiety. These findings may expand previous studies, with several theoretical and practical implications.

## 1. Introduction

The adolescents of today are a completely new generation, growing up in a digital environment. There is no question that the Internet has significantly changed the model of social interaction, especially for adolescents who have never known a world without the Internet [1]. Recently, social network sites (SNSs) have become part of the daily routine for adolescents. According to statistical data from the China Internet Network Information Center [2], adolescents have become the main group of SNSs users (at 34.5%). Using SNSs, they can share what they do and communicate with family and friends, to build and strengthen relationships. The social and psychological effects of SNS use on adolescents have been a focus of relevant researchers, as adolescence is a period of developing intimate relationships and expanding social networks [1]. The interactive and editable nature of SNSs makes adolescents more interested in self-disclosure on SNSs, which is one of the common ways they develop and maintain relationships. Utz indicated that positive and entertaining self-disclosure on SNSs increased the feeling of connection with friends [3]. Recently, researchers have focused on the effects of SNS-specific self-disclosure, rather than on SNS self-disclosure in general [4,5]; for example, the effect of the valence of self-disclosed content on Facebook on interactions with other individuals [6]. Therefore, there is a growing tendency for researchers to focus on how the use of SNSs affects adolescents’ physical and mental development, especially the specific self-disclosure behaviors seen commonly.

As adolescents begin to be independent of their parents and spend more time with peers, developing peer relations, especially close friendships, has become a vital task for adolescents, which could contribute to their psychological adaption and development [7]. At the same time, forming and strengthening social relations is a fundamental purpose of SNS users, and SNSs provide a handy way for adolescents to maintain and develop relationships with their offline friends [8]. Research indicates that using SNSs, especially disclosing and expressing personal information on SNSs, can strengthen social ties and enhance closeness and connectedness between users and friends or acquaintances [4,5].

Self-disclosure is a multi-dimensional variable. Previous research mainly focused on general SNS use and self-disclosure on SNSs (the amount of self-disclosure or the depth and width of disclosure) [5,9]; however, the valence of the self-disclosure should be paid attention to. In SNSs, positive self-disclosure is a more usual self-disclosure valence. For example, Facebook users care about impression management and can edit posts strategically, which will make them show more social attractiveness and trigger more public reactions [6]; thus, SNS users are more inclined to reveal positive aspects of their lives [10]. Therefore, based on relevant studies and real-life adolescents, this study focused on the positivity of self-disclosure and its association with adolescents’ friendship quality, as well as on the potential mediating and moderating roles (positive feedback and social anxiety) underlying this relation, which could expand on previous studies.

### 1.1. Positive Self-Disclosure on SNSs and Friendship Quality

Previous research suggests that communication in SNSs has an effect on offline social relationships. The primary and dominant motivation for adolescents’ use of SNSs, especially disclosing and expressing one’s information on SNSs, is to keep a connection with offline social relations and maintain offline relationships [11]. There are two opposite hypotheses for explaining the relationships between SNS use and offline friendships. The displacement hypothesis suggested that online communication has a negative effect on current offline friendships [12], while the stimulation hypothesis suggested that time spent online communicating with friends can enhance the quality of those relationships [12]. These two hypotheses were supported by various empirical studies. Previous researchers found that SNS use is negatively related to offline social relationships [13], whereas some recent evidence supported that SNS use enhanced closeness, connectedness and intimacy in offline friendships [14]. These inconsistencies may be due to different Internet activity choices, which should be further examined.

Disclosing personal information and emotion on SNSs had positive effects on the development of social relationships [15]. Self-disclosure is a common activity on SNSs, which is defined as the process of expressing and revealing personal information (thoughts, emotions and experiences) to others [16]; it is also a dominant factor for the development of offline friendships [3,14,15]. Adolescents could disclose personal information and experiences in different ways on SNS; for example, by posting photos, updating profile information, leaving comments, and a series of other online activities to control what they want to express [17]. In face-to-face communication, self-disclosure is the first step and main factor for intimate relationship development [18]. Researchers suggested that self-disclosure enhanced intimacy and satisfaction in social relationships offline [4].

Self-disclosure is defined as the process of expressing and revealing personal information (thoughts, emotions and experiences) to others [16]. Most status updates and profile information are positive experiences rather than intimate topics. They are defined as positive self-disclosure (compared to real self-disclosure), referring to the phenomenon that individuals tend to disclose positive information about themselves [3]. Adolescents can easily post/update their status, upload photos, and publish posts to express themselves positively and present a positive individual image, which is common on SNSs. Hence, positivity is a fundamental dimension to describe self-disclosure on SNSs [19]; however, most previous research focused mainly on general disclosure, or the quantity rather than the positivity of disclosure on SNSs [5,9,15]. Capitalization theory indicates that sharing positive events is a capitalization process. Positive events occur more often than negative events; thus, in offline social interactions, the majority of people disclose positive events, which could derive positive relational outcomes, including satisfaction, intimacy, closeness and trust in friendships [20]. Experimental evidence also supports that in online social interactions, sharing positive experiences strengthened the relationship between the expresser and the interactor [6]. Hence, the positivity of self-disclosure is more important in understanding SNSs’ positive influence on adolescents’ friendship quality. 

### 1.2. Positive Feedback as a Mediator

In face-to-face communication, self-disclosure not only has a direct effect on relationship development, it also has indirect effects on relationships via other social factors. In particular, SNSs provide an opportunity for users to receive social feedback in the forms of “Likes” and “Comments” on their status and profiles, and these signal acceptance, affection and attention [21]. When adolescents’ positively self-disclose in SNSs by posting photos, posts, personal information changes, etc., friends or family members evaluate and give feedback on these contents. Positive feedback indicates agreement, sympathy, friendliness and involvement; it could also contain self-disclosure by the poster’s friends and start conversations between adolescents and their friends [19]. Social feedback could also provide adolescents with emotional support and informational support, and it is a necessary condition for building strong social ties [22]. Research also found a mediating role of positive feedback in SNS use and psychological outcomes [23,24]. For example, Liu and Brown found positive feedback mediated the relationship between self-disclosure on SNSs and bonding social capital among Chinese college students [22]. Based on existing research, we continue along this line of thought that positive self-disclosure leads to more positive feedback.

At the same time, positive feedback may also be one of the facilitating factors for offline friendship quality. The interpersonal process model of intimacy assumes that, in offline communication, intimate relationships are developed in a dynamic process [18]. An individual discloses personal information, and then receives feedback from others, understands and interprets this feedback, and then improves their relationship development. In this model, perceived social feedback from friends is a main mediating factor in the relationship between self-disclosure and relationship maintenance. In view of this, the positive feedback perceived by adolescents on SNSs may have a positive impact on friendship quality. Similarly, capitalization theory also indicates that active responses from listeners are a mediator linked to positive events disclosure to affect the cognition of social relations in offline interactions [25]. Moreover, an experimental study demonstrated that partners’ response was an important mediator in the relationship between self-disclosure and friendship development [26]. According to the interpersonal process of intimacy, capitalization theory and related empirical findings, in this study, we hypothesized that positive feedback is a mediator of the relationship between self-disclosure on SNSs and the quality of friendships offline.

### 1.3. Social Anxiety as a Moderator

It is possible that online communication and its benefits to social outcomes could be moderated by individual differences, such as social anxiety [27,28]. The roles of individual differences on the impact of SNS use on social outcomes is still an undeveloped research area. In online social communication, there are two opposing theories that explain individual differences in acquiring benefits from SNS interactions. The ‘rich-get-richer’ hypothesis suggests that SNSs are just a new platform for social interaction, thus individuals with higher social competence and skills can obtain more benefits from SNSs. However, the social compensation hypothesis indicates that SNSs are not an extension of the face-to-face environment; the shortage of non-verbal cues and more control of personal information cater to individuals who lack social competence [29], especially socially anxious adolescents. Many researchers have conducted empirical studies based on different theoretical assumptions. Previous research found positive self-disclosure on SNSs is positively related to more social responses, but these results only appear in low self-esteem individuals [30]. Positive self-disclosure on SNSs is also related to more ‘Likes’, but this result only exists in the high social anxiety group [19]. Seo, Kim and Yang found that frequent social interactions on Facebook make people feel supported by friends, but this impact was stronger for the high interpersonal awareness group [31]. Therefore, based on the “rich get richer” hypothesis and previous research, we hypothesize that social anxiety is negatively moderated between positive self-disclosure and positive feedback, and that as adolescents’ social anxiety levels increase, positive self-disclosure results in less positive feedback.

Adolescents with high social anxiety lack social skills and often show avoidance and distress in social situations. Research suggests that high social anxiety interferes with positive communication offline. This may be because people with high social anxiety have more negative cognitive patterns [32,33]. High socially anxious individuals have interpretation bias when they communicate with others, which means highly anxious people tend to interpret other peoples’ social responses, especially ambiguous behavior and information, as negative and aggressive signals [34]. In SNSs, “Like” and “Comment” are the main and direct social feedback received by posters; these responses that lack some social cues may deliver ambiguous information and emotion. Posters have various interpretations of those responses, and it can be influenced by their cognition patterns [19]. Under the assumption of the “rich get richer” hypothesis, adolescents with high social anxiety may interpret positive feedback as invalid and negative, due to passive cognitive patterns, and this may negatively affect friendship quality. Therefore, we hypothesized that social anxiety negatively moderates the relationship between positive self-disclosure on SNSs and their offline friendship quality.

### 1.4. The Present Study

**Figure 1:**
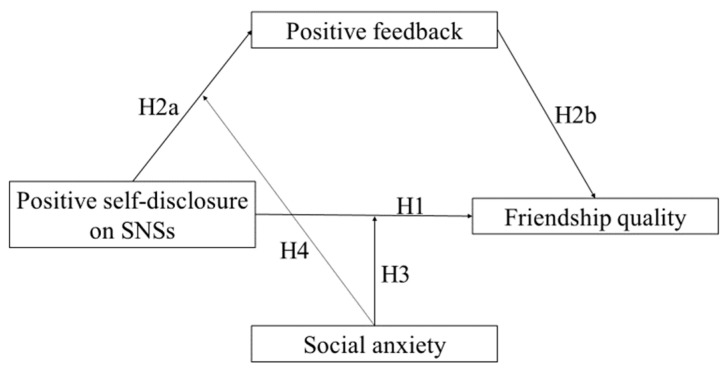
Research framework.

To summarize, this study aimed to examine the relationship between Chinese adolescents’ positive self-disclosure on SNSs and friendship quality and the mechanisms underlying this relationship, using a moderated mediation model (see Figure 1). Based on the research reviewed above, we designed this study to test the following hypotheses:

**H1:**  *Positive self-disclosure on SNSs could be positively associated with adolescents’ friendship quality offline.*

**H2:**  *Positive feedback could mediate the relationship between positive self-disclosure on SNSs and adolescents’ friendship quality.*

**H3:**  *Social anxiety could moderate the relationship between positive self-disclosure and friendship quality.*

**H4:**  *Social anxiety could moderate the relationship between positive self-disclosure and (perceived) positive feedback.*

## 2. Materials and Methods

### 2.1. Participants

A total of 1780 adolescents were voluntarily recruited from two public middle schools in Wuhan, a city in central China; and they were invited to complete a set of scales. Participants in the survey were voluntary, and their information was confirmed to be kept private. Finally, 1713 participants completed the survey without missing data (response rate = 96.24%). The age of the participants ranged from 11 to 19 years old, with a mean age of 14.64 years (*SD* = 1.76), and 49.2% were males.

### 2.2. Measurements

#### 2.2.1. Positive Self-Disclosure on SNSs

We use the positive self-disclosure on SNSs scale revised by Hollenbaugh and Ferries [35]. This seven-item (e.g., I usually disclose positive experiences of me on SNSs) measure asked subjects to indicate how much they agree on a scale from 1 (‘strongly disagree) to 5 (‘strongly agree’), including three reverse-coded items. When scores are averaged, high scores reflect participants’ disclosure on SNSs being more positive. The estimated value of Cronbach’s alpha reliability of this scale was 0.82.

#### 2.2.2. Friendship Quality

Adolescents’ friendship quality was assessed using the revision of the friendship quality scale [36]. A representative item is the following: “My friends understand me.” Participants were asked to rate each of the 14 items on a 7-point Likert-type scale, from completely untrue to completely true. The average score was used to evaluate the trust and reciprocal nature of friendship with general friends offline. An item that reduces the internal consistency reliability was removed [36]. The estimated value of Cronbach’s alpha reliability for the remaining 13 items in this research was 0.93.

#### 2.2.3. Positive Feedback

A positive feedback scale created by Liu and Brown was used to assess how often participants received positive feedback on their posts [24]. Representative items included the following: “How often they receive positive feedback on social networking sites from posting photos, opinions, and comments about their problems or things that made them proud.” Five items, which were answered using a 5-point Likert scale, showed a high Cronbach’s alpha reliability in this study (α = 0.86).

#### 2.2.4. Social Anxiety

The social anxiety scale created by La Greca and Lopez was used to test adolescents’ social anxiety levels [37]. Participants were asked to rate each of the 5 items (e.g., I worry about what other kids think of me) on a 5-point Likert scale, from completely disagree to completely agree. The items formed a high Cronbach’s alpha reliability in this study (α = 0.75).

#### 2.2.5. Control Variables

Given that previous research suggested that gender and age affected adolescents’ friendship quality [38], gender and age were included as control variables in this study.

### 2.3. Data analysis

SPSS 25.0 was adopted to sort and analyze the research data. Firstly, descriptive statistics and Pearson correlations were conducted for the main variables. Secondly, the SPSS macro PROCESS (model 8, which fit well with the hypothesized model) was adopted to test the hypothesized model, which was developed and widely used to test complex models with mediating and moderating roles; all the analyses were conducted using a 5000 bootstraps sample to generate 95% bias-corrected confidence intervals (CI) for all the indexes; if zero was not included in the 95% CI, the effects were regarded as significant.

## 3. Results

### 3.1. Preliminary Analyses

Table 1 presents the means and standard deviations, as well as the intercorrelations between the measures. There were significant intercorrelations between the measures, except for positive feedback and social anxiety.

### 3.2. Testing for Moderated Mediation

We expected that social anxiety would moderate the direct and indirect association between positive self-disclosure on SNSs and friendship quality via positive feedback. To test this moderated mediation hypothesis, we used the approach suggested by Muller, Judd and Yzerbyt [39]. Specifically, we estimated parameters for three regression models. In Model 1, we estimated the moderation effect of social anxiety on the relationship between positive self-disclosure and friendship quality. In Model 2, we estimated the moderation effect of social anxiety on the relationship between positive self-disclosure and positive feedback. In Model 3, we allowed both the partial effect of positive feedback on friendship quality, and the residual effect of positive self-disclosure on friendship quality, to be moderated by social anxiety. By the way, combined with the results of the three models (especially Model 3), the mediating effect could also be inferred through the predictive effect of positive self-disclosure and positive feedback on friendship quality. The specifications of these models can be seen in Table 2. In each model, we also controlled for relevant covariates. All the predictors were standardized to minimize multicollinearity [38]. For the purposes of our study, moderated mediation was established if either or both of two patterns existed [39]: (a) the path from positive self-disclosure on SNSs to positive feedback was moderated by social anxiety, and/or (b) the path from positive feedback to friendship quality was moderated by social anxiety.

**Figure 2:**
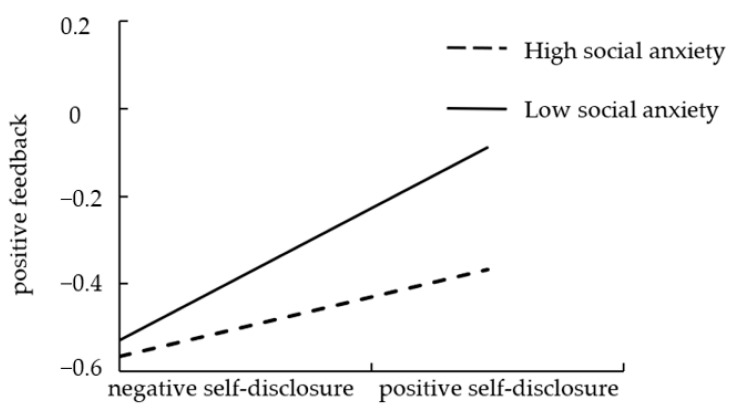
Social anxiety as a moderator of the relationship between positive self-disclosure and positive feedback.

As Table 2 illustrates, in Model 1 we observed an overall effect caused by positive self-disclosure on friendship quality (*B* = 0.36, *p* < 0.01) that was not moderated by social anxiety (*B* = −0.05, *p* > 0.05). Thus, hypothesis 3 was not supported. As Model 2 indicates, the main effect caused by self-disclosure positivity was significant (*B* = 0.23, *p* < 0.01), and the interaction between self-disclosure positivity and social anxiety was significant (*B* = −0.09, *p* < 0.05). At the same time, the mediating effect of positive feedback was also significant. To further examine the moderating effect, we plotted positive feedback against positive self-disclosure, separately for low levels of social anxiety and high levels of social anxiety (1SD below the mean and 1SD above the mean, respectively) (see Figure 2). The simple slope test indicated that for lower social anxious adolescents, higher positive self-disclosure was associated with higher positive feedback (*B_simple_* = 0.30, *t* = 7.97, *p* < 0.001). However, for higher socially anxious adolescents, the effect of positive self-disclosure on positive feedback was weaker (*B_simple_* = 0.14, *t* = 3.22, *p* < 0.005). Thus, social anxiety was a mediator for the association between positive self-disclosure and positive feedback.

Finally, we used SPSS Process to test the mediation effect under different conditions. For adolescents with low social anxiety, positive self-disclosure had a positive effect on friendship quality through positive feedback (*B* = 0.04, *SE* = 0.01, 95% CI = [0.02, 0.06]), and the mediation effect size was 9.46%. This effect was weaker in the group of high socially anxious adolescents (*B* = 0.02, *SE* = 0.01, 95% CI = [0.01, 0.03]), and the mediation effect size was only 4.60%. Thus, hypothesis 4 was fully supported.

Overall, the positive association between positive self-disclosure on SNSs and friendship quality was mediated by positive feedback, and the indirect effect of positive self-disclosure on friendship quality via positive feedback was moderated by social anxiety.

## 4. Discussion

Adolescents today are growing up in a digital environment, and with the popularity of SNSs in the information society, they have been a major user group of social media [2]. SNSs have become an essential platform for online interactions among adolescents, who share their lives and build and strengthen social relationships. Adolescence is a critical period for developing intimate relationships and expanding social networks [1]. With gradual independence from parents, friends play an increasing role in the social development for adolescents, and friendship becomes an important part of their social life. The editable nature of information and user interactions that SNSs have makes adolescents inclined to engage in positive and interesting self-disclosure, which will enhance their connections with friends [3], and the latter makes adolescents more concerned about the feedback received after self-disclosure. In addition, based on the “rich get richer” hypothesis and previous studies, we suggest that adolescents with different levels of social anxiety may perceive different levels of positive feedback and friendship quality during this process. Therefore, this study investigated the relationship between positive self-disclosure on SNSs and adolescents’ friendship quality, and the mediating effect exerted by positive feedback and the moderating effects exerted by social anxiety.

### 4.1. Direct and Indirect Effects

We found that positive self-disclosure on SNSs had a direct predictable effect on adolescents’ friendship quality offline. Self-disclosure is one of the most important tools for adolescents to build trusting relationships [4]. SNSs are very convenient platforms for the digital generation to build and strengthen social relationships [8], and they often self-disclose on SNSs. Moreover, for impression management purposes, social media users show positive self-disclosure compared to real self-disclosure [10]. This finding was consistent with intimacy as a process model and capitalization model, as well as with previous research. Utz found that positive self-disclosure on SNSs can enhance perceived connection among social relationships [3]. On SNSs, the positive effect of the positive tendency of self-disclosure on friendship quality may also be explained by the increase in personal attraction. In addition, disclosing more positive events induced positive self-perception in adolescents, and then influenced their social adaptation [24]. Thus, while we are concerned about offline friendship quality during adolescents’ growth, we also need to care about positive self-disclosure on SNSs, which is important for adolescents’ social adaptation development.

In the present study, we also found that positive self-disclosure on SNSs was positively associated with positive feedback from friends, which was, in turn, related to the enhancement of friendship quality. The results confirmed our hypothesis, and added to the limited literature on positive feedback as an underlying mechanism that accounts for the association between self-disclosure on SNSs, in particular, positive self-disclosure and friendship among adolescents [3].

The interpersonal process model of intimacy indicated that, in offline social interaction, self-disclosure was the origin of intimate relationship building, and the perception of positive feedback also exerted a crucial role in intimate relationship maintenance and future development [18]. The convenience of SNSs makes positive self-disclosure a common phenomenon, and the interactive nature makes feedback an important factor in enhancing interpersonal connections. When individuals update their personal status on SNSs, friends give positive feedback on the relevant content, which will reveal attention and concern for the poster, which in turn maintains and strengthens friendships. Moreover, on SNSs, previous studies indicated positive feedback had a mediating role in online self-disclosure and positive social outcomes [24,30]. In the present study, the results confirmed our mediation hypothesis, and were consistent with the limited previous studies available. It also provided new evidence of capitalization theory in online social communication.

In addition to the overall mediation result, each of the individual links in our model is noteworthy. First, we found that more positive self-disclosure on SNSs was related to more positive feedback, which was consistent with previous findings [19,24]. For example, große Deters, Mehl and Eid [19] found that positive self-disclosure had a positive effect on receiving more ‘Likes’ from friends. Second, the positive relation between positive feedback and friendship quality was also consistent with findings in the literature. Previous research had indicated that positive feedback contributed to an increase in bonding social capital [24], and not receiving expected likes might lower the feeling of connection with friends [3]. At the same time, this finding also expanded previous studies, and deepens our understanding of the influences of SNS use by focusing on the specific activity on SNSs (positive self-disclosure) and an important development indicator—friendship quality.

### 4.2. Moderating Effect

Another purpose of the present study was to explore the moderating role of adolescent social anxiety traits in the relationship between positive self-disclosure and friendship quality, and between positive self-disclosure and positive feedback. As mentioned earlier, adolescents who have grown up entirely in the digital era prefer self-disclosure on SNSs to strengthen their friendships, and prefer to present positive messages related to themselves for impression management purposes. The process may vary due to differences in adolescents’ individual characteristics. Since digital natives prefer to communicate in cyberspace and lack actual social communication experience, they are prone to anxiety during communication, making social anxiety one of the factors that has received wide attention in recent years. Based on the “the rich get richer” hypothesis, individuals with higher social competence are more likely to benefit from social media. It has also been found that social anxiety moderates the relationship between online communication and social outcomes [27,28]. Therefore, we argue that the beneficial effects of positive self-disclosure on the quality of offline friendship quality diminish as the level of social anxiety increases among adolescents. We found that social anxiety did not have a statistically significant moderating effect on direct relationships, though the effect of positive self-disclosure on friendship quality was a little stronger in the low socially anxious group than the high anxiety group. Hypothesis 3 was not supported, but the trend still supported the ‘rich-get-richer’ hypothesis [40], which means that for low socially anxious adolescents, they would benefit more from SNS communication, particularly when they use SNSs to maintain their offline friendships.

As predicted, the moderated mediation effect was found. The positive feedback had a moderating role in the mediating model, particularly in the relationship between positive self-disclosure and positive feedback, especially in the first path. As individuals with high social anxiety are more prone to negative cognitive patterns [32,33] and tend to interpret others’ reactions as negative messages or signals of aggression [34], this may lead to reduced positivity they perceive from their friends’ feedback. Compared with low social anxiety adolescents, the predictable effect of positive self-disclosure on positive feedback was weaker in the high social anxiety group. This result was consistent with previous research, which found that social anxiety was a moderator in the relationship between positive status updates and the amount of ‘Likes’ [19].

Firstly, the moderated mediation result could be explained by the interpretation bias of the social anxiety group. Due to a lack of a sense of safety, some adolescents tend to interpret friends’ responses as invalid and negative, which in turn, is detrimental to friendships with their friends [18]. Researchers had confirmed that cognitive bias, especially interpretation bias, existed in high social anxiety people [33]; thus, they are more likely to interpret positive feedback, especially feedback received via SNSs, an ambiguous environment [34], as invalid and negative [34]. They tended to think that positive social activity also has some substantial threats to their own and their relationship with others, so they cannot benefit as much as the low anxiety group [41]. Secondly, fearing others’ evaluation can help us to understand the result. As self-presentation theory showed, high social anxiety people are fearful of negative evaluations from friends [42]. In addition, Weeks, Heimberg and Rodebaugh found that the high anxiety group also feared positive evaluations and responses because they worried those positive feedbacks would be replaced by negative responses, which was harmful to their friendship development [43]. Therefore, we should pay more attention to how adolescents with high social anxiety are affected in the process of using SNSs, and guide them to correctly understand online information, develop healthy friendship relationships, and thus promote their social development.

## 5. Conclusions

The present study focused on positive self-disclosure on SNSs, rather than general SNS use and self-disclosure, and revealed the relationship between positive self-disclosure and friendship quality among adolescents, as well as the underlying mechanism. The findings in this study added some new evidence to research the relationship between SNS use and social relation development; moreover, we applied offline intimate relationship theory, such as intimacy as an interpersonal process and capitalization theory, to SNS social interactions, to expand our understanding of the influences of SNS use by focusing on a specific activity, and the influencing factors of adolescents’ friendship quality. Furthermore, the findings in the present study provide some empirical references to improve adolescents’ friendship quality in the current information age—first, adolescents should reasonably use SNSs to achieve good influences, and good social experiences (such as positive feedback being beneficial) should be encouraged; then, socially anxious adolescents should be paid much attention to.

However, this study has some limitations. Firstly, we use self-reported data to examine online behavior. Future research can add crawled data from SNSs and compare it with self-reported data, in order to examine online behavior from a different perspective. Secondly, we used positive feedback as a mediator and only focused on its frequency. Different types of feedback, such as ‘Likes’ and ‘Comments‘, as well as the amount and time of feedback can be added in future studies. Thirdly, we used a cross-sectional design, but this method cannot reveal the cause-and-effect relationship between positive self-disclosure and friendship quality. Thus, a longitudinal design should be applied to future research, in order to reveal the cause-and-effect relationship between self-disclosure on SNSs and friendships among adolescents. Finally, more factors (such as grade) should be considered in future studies, and other potential mechanisms and potential individual differences (e.g., gender difference) should be further examined as well; the influences of positive self-disclosure should be comprehensively discussed, due to the fact that sharing positive experiences may induce the upward social comparison, and may further lead to deleterious outcomes [20].

## Figures and Tables

**Table 1:** Means, standard deviations and intercorrelations of the measures.

Variables	*M* ± *SD*	1	2	3	4
1 positive self-disclosure	3.57 ± 0.73	1			
2 positive feedback	2.61 ± 0.92	0.19 **			
3 friendship quality	5.53 ± 1.02	0.27 **	0.16 **		
4 social anxiety	3.05 ± 0.88	−0.13 **	−0.04	−0.12 **	1

** *p* < 0.01.

**Table 2:** Regression results for the moderated mediation model.

	Model 1 (Criterion FQ)			Model 2 (Criterion PF)			Model 3 (Criterion FQ)		
	*B*	*SE*	95% CI	*B*	*SE*	95% CI	*B*	*SE*	95% CI
X: VSD	0.36 **	0.04	(0.28, 0.43)	0.22 **	0.03	(0.16, 0.30)	0.33 **	0.04	(0.26, 0.40)
MO: SA	−0.10 **	0.03	(−0.16, −0.04)	−0.02	0.03	(−0.07, 0.04)	−0.10 **	0.03	(−0.16, −0.04)
XMO: VSD × SA	−0.05	0.04	(−0.13, 0.03)	−0.09 *	0.04	(−0.17, −0.02)	−0.03	0.04	(−0.11, 0.05)
ME: PF							0.12 **	0.03	(0.06, 0.18)
MEMO: PF × SA							−0.01	0.03	(−0.09, 0.06)
CO: Gender	0.13 **	0.05	(0.04, 0.23)	0.06	0.04		0.12 *	0.04	(0.03, 0.22)
CO: Age	−0.04	0.01	(−0.07, −0.02)	0.03 **	0.01		−0.05 *	0.01	(−0.08, −0.02)
*R^2^*	0.09			0.05			0.10		
*F*	30.29 **			12.42 **			26.52 **		

* *p* < 0.05. ** *p* < 0.01.. CO = control variable; X = independent variable; MO = moderator; XMO = interaction between independent variable and moderator; ME = mediator; MEMO = interaction between mediator and moderator; VSD = positive self-disclosure; PF = positive feedback; SA = social anxiety; FQ = friendship quality.
